# Prostate carcinoma with neuroendocrine differentiation successfully treated by early detection with imaging examination

**DOI:** 10.1002/iju5.12219

**Published:** 2020-09-14

**Authors:** Momoko Kobayashi, Masahiro Hagiwara, Hideyuki Yamashita, Makoto Ohbu, Akira Irie

**Affiliations:** ^1^ Department Urology Kitasato InstituteHospital, Kitasato University Minato‐ku Tokyo Japan; ^2^ Department Pathology Kitasato Institute Hospital, Kitasato University Tokyo Japan

**Keywords:** immunohistochemistry, neuroendocrine carcinoma, prostate cancer, prostate‐specific antigen, prostatectomy

## Abstract

**Introduction:**

Clinicopathological features of neuroendocrine differentiation of prostate carcinoma have not been totally clarified yet. It is known to be associated with poor prognosis.

**Case presentation:**

A patient with growing prostate‐specific antigen was diagnosed with prostate carcinoma and treated by laparoscopic prostatectomy. The pathological examination revealed the neuroendocrine differentiation of the tumor. Early detection of prostate carcinoma with neuroendocrine differentiation can be difficult due to its low expression of prostate‐specific antigen. The imaging examination contributed to the early detection. In the follow‐up period of 2 years, the patient remains cancer free.

**Conclusion:**

Recently, the treatment options for prostate carcinoma have been expanded. Precise assessment of immunohistochemical nature of the tumor may be helpful for individualized decision‐making.

**Abbreviations & Acronyms**
EPE: extraprostate extension
HE: hematoxylin and eosin
MRI: magnetic resonance imaging
NED: neuroendocrine differentiation
PSA: prostate‐specific antigen
TRUS: transrectal ultrasound

**Keynote message**

NED of prostate carcinoma is a diagnostic challenge due to its low expression of PSA. Additionally, the resistance to chemo‐, radio‐, and hormonal therapy narrows the therapy options. We experienced a case of prostate carcinoma with primary NED which was successfully treated by surgery. Imaging examination was helpful for early detection of the carcinoma, and immunohistochemical assessment may contribute to individualized decision‐making.

## Introduction

Focal NED of prostate carcinoma has been reported as a rare variant.^1^ NED is known for its resistance to conventional hormone therapy, radiotherapy, and chemotherapy and is therefore considered to be a negative prognostic factor.^2^, ^3^ Early detection of these tumors might be required in regard. Despite the frequency and possible impact on clinical outcome, there is no consensus on its management. We present a case of prostate carcinoma with NED successfully treated by surgery.

## Case presentation

**Fig. 1:**
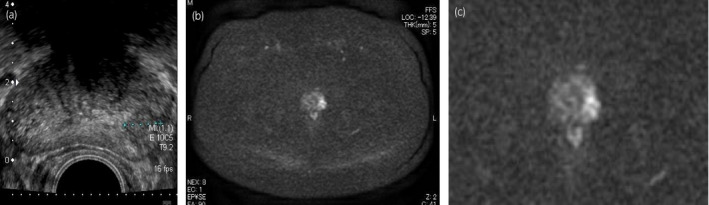
Imaging of the prostate. (a) TRUS of prostate, left peripheral zone suspicious for prostate carcinoma. (b) MRI of prostate, DWI, left peripheral zone suspicious for prostate carcinoma. (c) MRI of prostate, DWI, left peripheral zone suspicious for prostate carcinoma, magnified.

A 69‐year‐old Asian man presented to the department of urology of our hospital due to increasing PSA, growing from 1.765 ng/mL in the previous to 2.680 ng/mL in the presenting year, pointed out in the annual checkup. We performed a TRUS: prostate volume was 28.4 mL and there was a clearly localized hypoechoic area in the left peripheral zone of the prostate (Fig. 1a). MRI revealed the suspicion for prostate carcinoma (T2 low, diffusion‐weighted MRI high) in the identical area as in the TRUS (Fig. 1b,c). He neither had family history of prostate, breast nor ovarian carcinoma.

Transrectal prostate needle biopsy was performed. The result was adenocarcinoma, Gleason score 4 + 4, in 3/14 cores. The clinical diagnosis was adenocarcinoma of the prostate, cT2aN0M0.

**Fig. 2:**
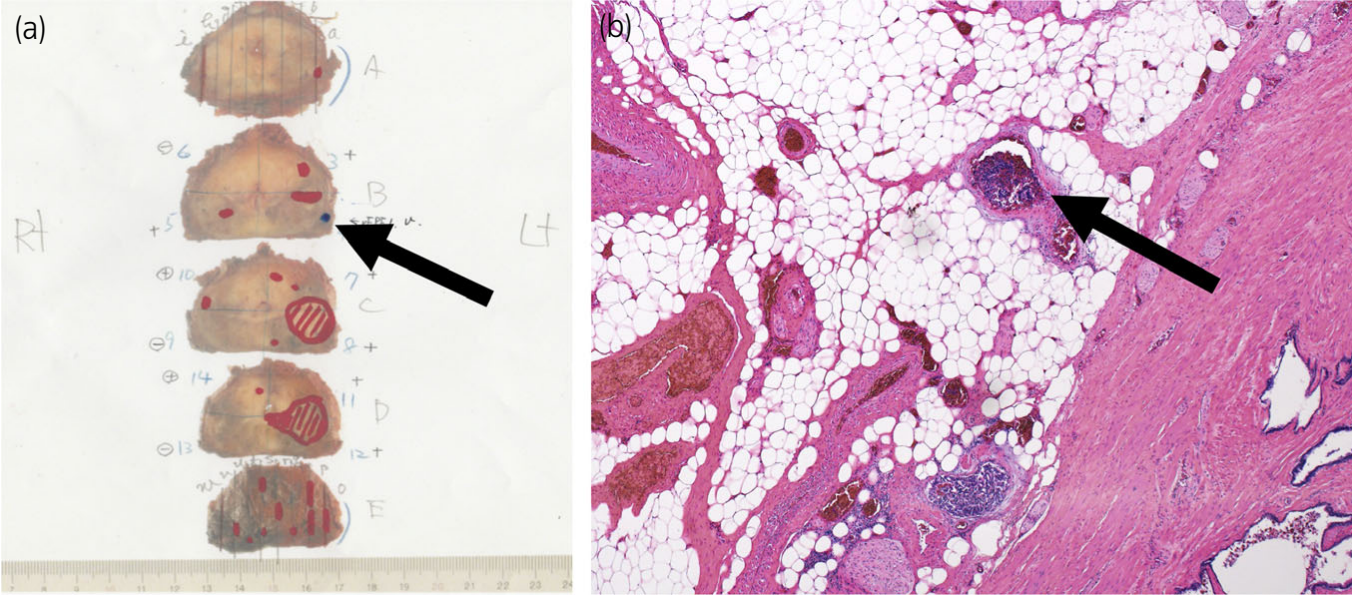
LRP specimen EPE. (a) Macroscopy, arrow (←) shows the site of EPE+. (b) HE, ×40, arrow (←) shows venous embolism of tumor cells in extraprostate adipose tissue.

Laparoscopic radical prostatectomy and left‐sided obturator lymph node dissection were performed. The pathological diagnosis was adenocarcinoma of the prostate with NED, Gleason score 4 + 3, ly0, v1, pn0, sv0, EPE1, RM0, N0(0/4), and IDC‐P (+). The main tumor, sized 20 mm, was located in the left peripheral zone as shown in the imaging (Fig. 2a). Venous invasion of tumor cells on the very left basal side of the tumor was evaluated as EPE positive (Fig. 2b).

**Fig. 3:**
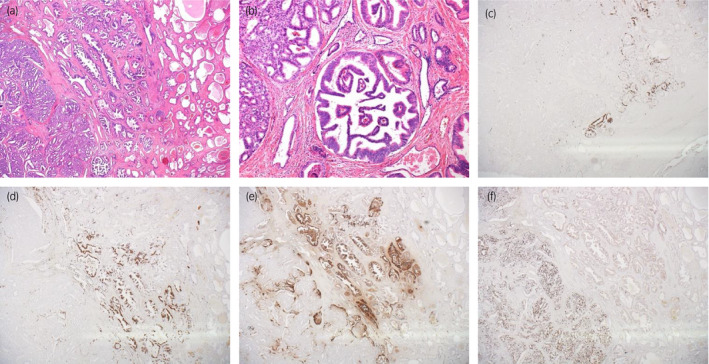
Prostate carcinoma, LRP specimen. (a) HE, ×40. (b) HE, ×400, rosette formation. (c) Synaptophysin, ×400. (d) CD56, ×400. (e) Chromogranin A, ×400. (f) Ki‐67, ×400.

HE staining showed clearly shaped rosette formations inside the dominant tumor nests. Despite the malignant pathology, necrosis and nuclear atypia were not prominent. These phenomena suggest NED of the tumor cells (Fig. 3a,b).

Consequently, we performed immunohistochemical staining with neural (synaptophysin, CD56), endocrine (chromogranin A), and proliferation markers (Ki67) (Fig. 3a–f). About 40% of the tumor were positive for the neuroendocrine markers. Ki67‐labeling index was high in the area without NED but low in the area with NED. Paradoxically, PSA staining was negative for the tumor but positive for the rest of the prostate.

**Fig. 4:**
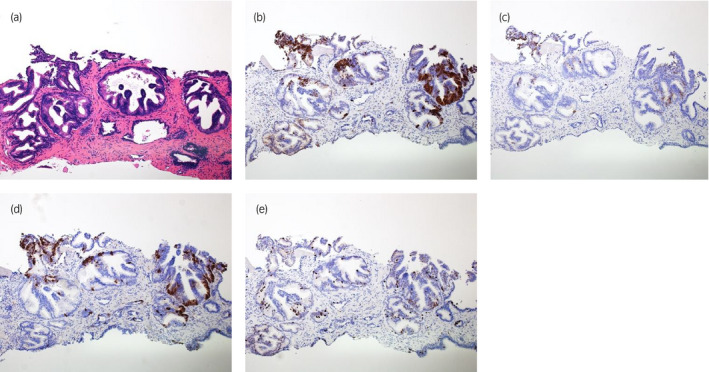
Prostate needle biopsy specimen. (a) HE, ×10. (b) Synaptophysin, ×10. (c) Chromogranin A, ×10. (d) CD56, ×10. (e) Ki‐67, ×10.

Retrospectively, we reviewed the biopsy specimen and did additional staining for neuroendocrine markers. In the biopsy specimen, the corresponding rosette formation could be detected. The immunohistochemical staining also showed identical results as in the surgery specimen, that is focal positivity for synaptophysin, chromogranin A, CD56, and Ki‐67 (Fig. 4a–e).

The patient has not received any adjuvant therapy. In the regular follow‐up, no recurrence nor metastasis have been detected and his postoperative PSA remained <0.009 ng/mL during the follow‐up period of 2 years.

## Discussion

Clinicopathological features of prostate carcinoma with NED have not been totally clarified yet. Helpap *et al*. reported NED as potent independent negative prognostic factor for untreated, clinically organ‐confined disease and intermediate grade tumor.^2^ There are several reasons explaining the poor outcome. First, growth‐stimulating factor produced by neuroendocrine cells promotes the proliferation of the tumor. Second, resistance to conventional hormone therapy, radiotherapy, and chemotherapy contributes to increased aggressiveness caused by androgen receptor deficiency. Third, it is difficult to make an early diagnosis due to the lack of reliable biomarkers, in contrast to typical adenocarcinoma.^2^, ^3^

In our case, PSA at the time of first visit counted 2.680 ng/mL, which is relatively low to suspect malignancy. Screening for prostate carcinoma usually includes PSA checkup, but imaging is not routinely recommended.^4^ Nevertheless, we decided to perform TRUS due to the PSA velocity. The TRUS and following MRI findings convincingly pointed to the existence of malignancy and we performed the prostate biopsy. This episode reconfirms the importance of imaging examination, especially for early detection of atypical types of prostate carcinoma.

Radical prostatectomy and radiotherapy are currently the main treatment options for organ‐confined prostate carcinoma depending on the risk category. Anti‐androgen agents are frequently applied additionally to radiotherapy as neoadjuvant and adjuvant treatments, and in case of recurrence after surgery or radiotherapy.^4^ In our case, the biopsy specimen was initially diagnosed as pure adenocarcinoma. Our patient belonged to a high‐risk group due to his high Gleason score.

In this case, recommended treatment options are radiotherapy, radiotherapy + androgen deprivation therapy, or radical prostatectomy. In contrast to pure adenocarcinoma of the prostate, NED is known for its resistance to hormone therapy and radiotherapy.^3^ Radiotherapy may have higher risk for treatment failure and surgery might have been the appropriate therapy in his case. Nowadays, the treatment options for prostate carcinoma are very complicated. Evaluating the character of the tumor precisely at the time of initial diagnosis may be helpful for deciding which treatment option to pursue.

At our institution, the biopsy specimen is usually stained with HE. In the presenting case, we additionally requested immunohistochemical examination which revealed the existence of focal NED changes. Retrospectively, atypical rosette formation can be seen even in the HE staining of the biopsy specimen. Screening all the biopsy specimens with neuroendocrine markers is neither reasonable nor recommended; however, in cases with unusual findings, such as low PSA and architectural/cytological anomalies in HE, immunohistochemistry may lead to a precise diagnosis. In 1994, Cohen *et al.* denied the correlation between the existence of NED in needle biopsy specimens and tumor progression. In their study, the object was limited to patients who had received radical prostatectomy.^5^ Whether NED in biopsy specimens may have an impact on prognosis if treated by other options has not been researched yet.

## Conclusion

Treatment options for prostate carcinoma have expanded drastically in recent years. Though, patients’ physical status, PSA, PSA doubling time, and Gleason score remain the markers for prediction of clinical outcome.^4^ Immunohistochemical findings rarely influence the initial decision‐making. Moreover, in many cases, the existence of NED is unknown at this timing. Further research of NED in biopsy specimens and its impact on clinical outcome in the current setting is interesting and will contribute to the precise assessment of the disease and more individualized decision‐making.

## Conflict of interest

The authors declare no conflict of interest.

## References

[1] Fine SW . Neuroendocrine tumors of the prostate. Mod. Pathol. 2018; 31: 122–32. 29297494 10.1038/modpathol.2017.164

[2] Helpap B , Köllermann J , Oehler U . Neuroendocrine differentiation in prostatic carcinomas: histogenesis, biology, clinical relevance and future therapeutic perspective. Urol. Int. 1999; 62: 133–8. 10529661 10.1159/000030376

[3] Hu C‐D , Choo R , Huang J . Neuroendocrine differentiation in prostate cancer: a mechanism for radioresistance and treatment failure. Front. Oncol. 2015; 5: 90. 25927031 10.3389/fonc.2015.00090 PMC4396194

[4] Mottet N , van den Bergh RCN , Briers E *et al* EAU‐ESTRO‐ESUR‐SIOG Guidelines on Prostate Cancer. 2018 [Cited 7 Jun 2019.] Available from URL: https://uroweb.org/wp‐content/uploads/EAU‐ESUR‐ESTRO‐SIOG‐Guidelines‐on‐Prostate‐Cancer‐large‐text‐V2.pdf

[5] Cohen MK , Arber DA , Coffield KS , Keegan GT , McClintock J , Speights VO Jr . Neuroendocrine differentiation in prostatic adenocarcinoma and its relationship to tumor progression. Cancer 1994; 74: 1899–903. 8082095 10.1002/1097-0142(19941001)74:7<1899::aid-cncr2820740712>3.0.co;2-u

